# Mechanical Thrombectomy in Two Children Under the Age of One Year

**DOI:** 10.1002/ccr3.71166

**Published:** 2025-10-15

**Authors:** Erensu Mengüşoğlu, Francesco Puccinelli, Steven D. Hajdu, Bruno Bartolini, Guillaume Saliou

**Affiliations:** ^1^ Department of Neurology Kartal Lütfi Kırdar City Hospital Istanbul Turkey; ^2^ Department of Diagnostic and Interventional Radiology Lausanne University Hospital (CHUV) Lausanne Switzerland

**Keywords:** infant, pediatric stroke, retinoblastoma, thrombectomy, thromboaspiration, thromboembolism

## Abstract

This case series examines mechanical thrombectomy (MT) with thromboaspiration in two infants under 1 year, a rarely performed procedure due to ethical challenges and limited data. Both cases involved thromboembolic complications during intra‐arterial chemotherapy for retinoblastoma. An 11‐month‐old with left posterior inferior cerebellar artery occlusion and a 5‐month‐old with basilar artery occlusion underwent MT, achieving full recanalization (eTICI = 3) without infarction or hemorrhage. Postoperative imaging confirmed no complications, and both patients had normal neurological outcomes (pedNIHSS = 0). These cases highlight the feasibility and safety of MT in infants, demonstrating its potential to achieve excellent outcomes in high‐risk situations. Further research is needed to establish standardized guidelines and expand MT's use in pediatric stroke management.

**Summary**

- Mechanical thrombectomy with direct thromboaspiration can be considered for infants under 1 year with thromboembolic complications.

## 1 Introduction

Ischemic stroke in childhood is rare, affecting 1–2 per 100,000 children annually [1]. It presents significant challenges due to the anatomical and physiological characteristics. As in adult patients, pediatric strokes, particularly in neonates and infants, require prompt and precise interventions to mitigate long‐term neurological deficits [2]. Mechanical thrombectomy (MT) is a widely accepted treatment for acute ischemic stroke in adults, offering benefit in terms of recanalization and functional outcomes. Pediatric strokes are rare and there are ethical considerations surrounding clinical trials in this vulnerable group. Due to limited evidence, especially in infants under 1 year, MT is not usually considered and remains a topic of ongoing research and debate.

To date, literature and case studies support the feasibility and benefit of MT in children, although comprehensive data and standardized guidelines are still lacking [3, 4, 5]. The Save ChildS [3] study provided encouraging results, indicating that thrombectomy could be safely performed in children, although it emphasized the need for larger prospective studies to validate the findings. Kossorotoff et al. discussed recanalization treatments and highlighted the necessity of specialized skills and techniques for performing MT in pediatric patients [4].

In this case report we present two extremely rare cases of mechanical thrombectomy (MT) performed on infants under the age of one who developed thromboembolic complications during intra‐arterial chemotherapy for retinoblastoma. The two cases illustrate the feasibility and challenges of performing MT in such young patients and contribute to the growing body of literature advocating for the further investigation of this procedure in pediatric stroke management.

## 2 Case History/Examination

We report two cases of thrombectomy in two children, aged 5 and 11 months at the time of intervention. In both, cerebral artery occlusion occurred during intra‐arterial infusion therapy for retinoblastoma and to date, they represent the only two such cerebral artery occlusions on angiography in our cohort of more than 1094 sessions of intra‐arterial chemotherapy infusion. During this intervention, our anticoagulation protocol involves 40 Units/kg injected intravenously at the beginning of catheterization, with an additional half‐dose injection every hour.

### 2.1 Case 1

**FIGURE 1:**
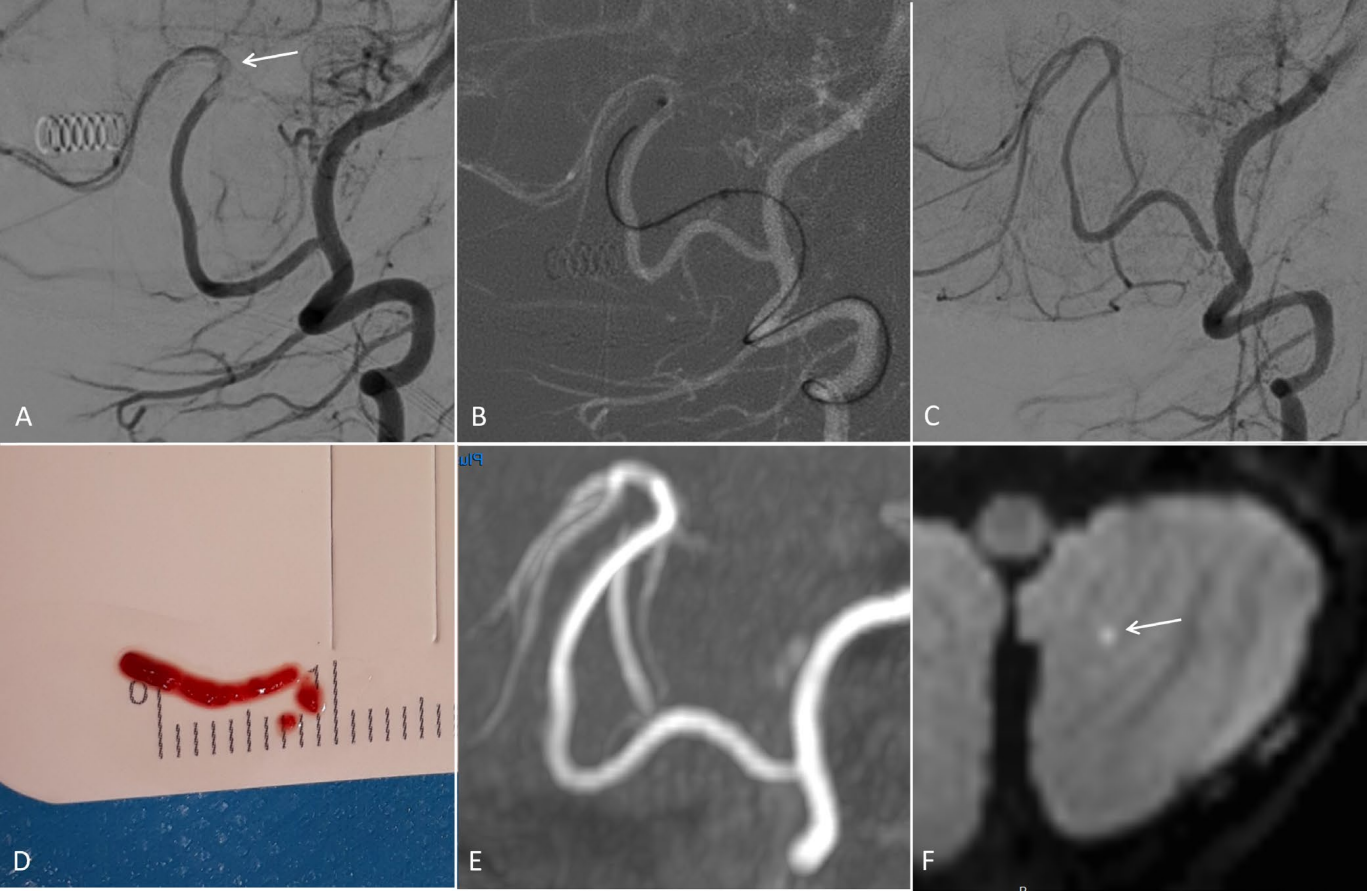
Patient 1, 11‐months‐old. Clot embolus in the PICA (A: Arrow) treated by direct thromboaspiration through a Phenom 0.027 microcatheter (B) with an eTICI score of 3 after 2 passes (C). The clot (D) was stuck to the tip of the microcatheter. MRA the day after (E) showed a patent PICA with only a small spot on DWI (F: Arrow). The pedNIHSS was 0 when the patient woke up after the intervention.

The first per‐intervention cerebral artery thromboembolic complication happened in an 11‐month‐old child, of 8 kg (Figure 1) treated for a right eye retinoblastoma stage C according to the International Intraocular Retinoblastoma Classification (IIRC). The patient didn't have risk factors for stroke, especially coagulation or platelet aggregation disorders related to systemic chemotherapy. Pre‐intervention physical neurological examination was normal, except for the loss of vision due to the oncological disease. During the second session of intra‐arterial chemotherapy infusion, an occlusion of the left Posterior Inferior Cerebellar Artery (PICA) occurred during left vertebral artery catheterization to access the left ophthalmic artery with a P‐com approach. We chose this approach because through the internal carotid approach, the microcatheter was unstable with back‐flow to the internal carotid and with no usable supply to the retina through the external carotid artery. We immediately did an intravenous injection of aspirin (5 mg/kg) in addition to heparinization and waited 30 min for signs of spontaneous recanalization. As the occlusion was unchanged, we decided to conduct a thrombectomy. We performed direct thromboaspiration through a Phenom 0.027 microcatheter (Medtronic, Tolochenaz, Switzerland) with a Synchro 0.014 guidewire (Stryker, Fremont, California, United States), pushed into the left PICA up to the clot through a Fargo mini 4F catheter (Balt, Montmorency, France) placed at the V4 segment in front of the origin of the PICA. We aspirated through the Phenom 0.027 for 2 min and then gently removed the microcatheter. The clot was caught at the tip of the microcatheter and removed in one piece with an eTICI = 3 after the second pass. The time from identification of cerebral artery occlusion to recanalization was 116 min.

### 2.2 Case 2

**FIGURE 2:**
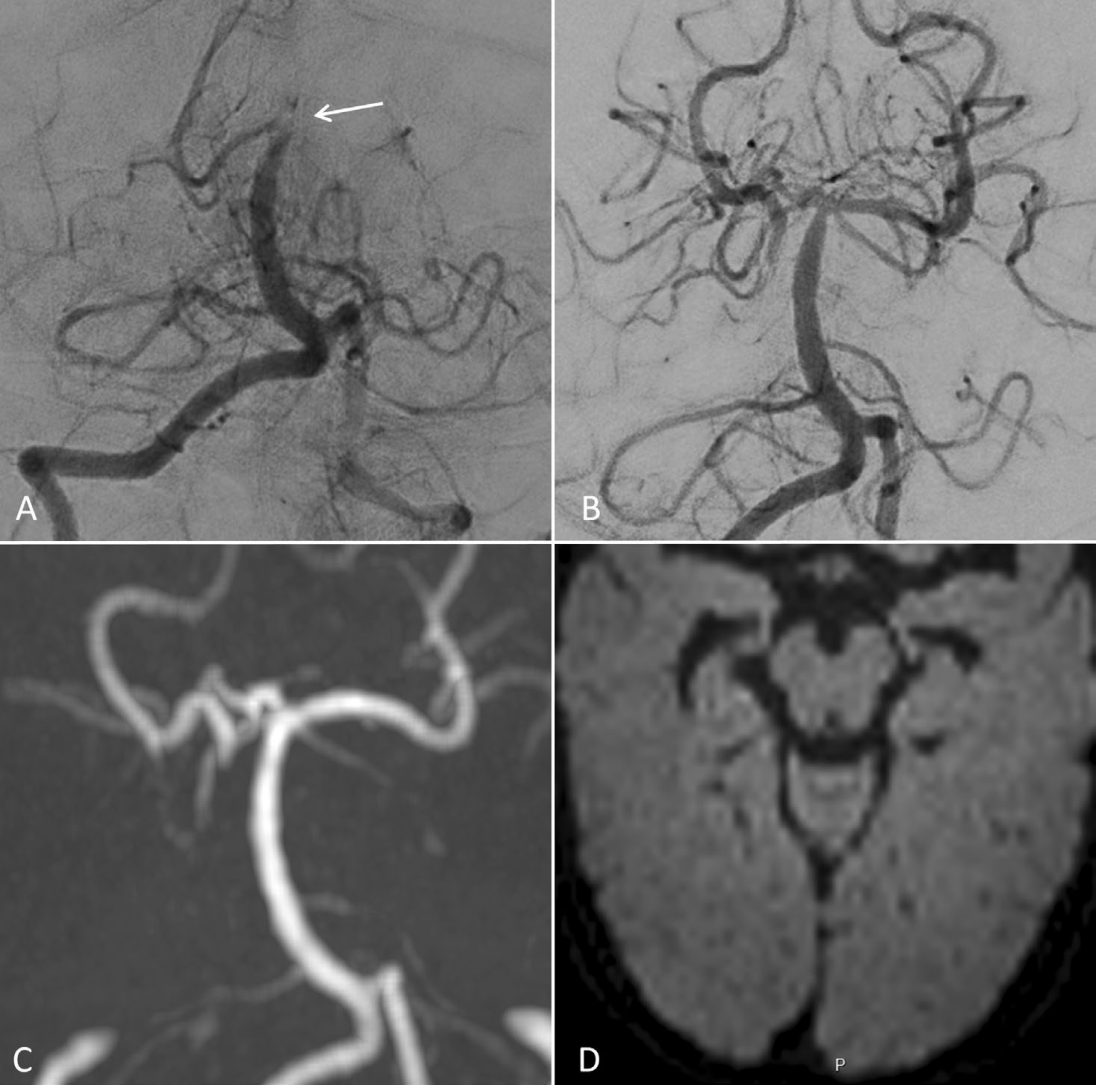
Patient 2, 5‐months‐old. Clot embolus at the top of the basilar artery (A: Arrow) treated by direct thromboaspiration through a Sofia 5F catheter with an eTICI score of 3 after 1 pass (B). MRA the day after (C) showed a patent basilar artery with no diffusion restriction on DWI (D). The pedNIHSS was 0 when the patient woke up after the intervention.

The second per‐procedure cerebral artery thromboembolic complication happened in a 5‐month‐old child, weighing 7.5 kg (Figure 2) treated for a left eye retinoblastoma stage D (IIRC). The patient didn't have risk factors for stroke, especially coagulation or platelet aggregation disorders related to systemic chemotherapy. Pre‐intervention physical neurological examination was normal, except for the loss of vision due to the oncological disease. During the first session of intra‐arterial chemotherapy infusion, an occlusion of the distal tip of the basilar artery occurred during left vertebral artery catheterization to access the left ophthalmic artery through a P‐com approach, used for the same reason as in case 1. We did a venous injection of aspirin (5 mg/kg) and immediately a direct thromboaspiration with a Sofia 5F catheter with a Synchro 0.014 guidewire, pushed into the basilar artery up to the clot. We performed direct aspiration through the Sofia 5F for 2 min and gradually removed the catheter. The clot was removed in one piece with an eTICI = 3 after one pass. The time from identification of the cerebral artery occlusion to recanalization was 41 min.

## 3 Outcome and Follow‐Up

The two babies were then kept under Aspirin 5 mg/kg for 6 weeks.

In Case 1, we did a further cerebral Magnetic Resonance Imaging (MRI) and Magnetic Resonance Angiography (MRA) the next day that demonstrated only a tiny spot of restriction on diffusion‐weighted imaging (DWI), without territorial infarction. There was no hemorrhage, especially focal subarachnoid hemorrhage (SAH). The neurological examination of the patient was normal (pedNIHSS = 0) at day one and at 6 months. At her follow‐up cerebral MRI, performed for routine check‐up to monitor her retinoblastoma at 6 months, there was no cerebral ischemia scarring in the posterior circulation territory, and the right PICA was normal.

For Case 2 we also repeated a cerebral MRI and MRA on the next day, which demonstrated no restriction on DWI, and no territorial infarction. There was no hemorrhage, especially focal SAH. The neurological examination of the patient was normal (PedNIHSS = 0) at day one, and 3 and 18 months. At her follow‐up cerebral MRI, performed for routine check‐up to monitor her retinoblastoma at 3 and 18 months, there were no cerebral ischemia scarring in the posterior circulation territory, and the basilar tip was normal.

## 4 Discussion

We reported two rare cases of thrombectomy by direct thromboaspiration in the cerebral artery of children of less than 1 year of age having cerebral artery thromboembolic complications after chemotherapy for retinoblastoma. Both cases were not the regular endovascular management of symptomatic stroke in children but complications under general anesthesia without assessment of the clinical status. Two other case reports in the literature detail the thrombectomy technique with a stentriever in a 6‐month‐old [6] and a 9‐month‐old [7], also without stroke symptoms at presentation. In pediatric case series investigating thrombectomy in the acute phase of stroke, the mean age at presentation was much older than in these two cases and in ours: over 10 years old, and in the meta‐analysis of Bilgin et al., no MT (either with thromboaspiration or stentriever) under 13 months of age [5] was reported. This is because stroke diagnosis in children is more often delayed or missed compared to adults, especially in children under the age of one. Indeed, in this young population, stroke symptoms are not specific, especially with seizures, once the parenchyma is necrotized [1].

An important result from our cases is that thromboaspiration was successful in both, indicating a potentially safe method despite the very small and weak arteries, and especially, no SAH occurred. We chose direct aspiration thrombectomy rather than the stentriever because, even though we haven't demonstrated a better effect, the risk of SAH appears lower in our experience, also supported by the literature [8, 9, 10]. Further, removing a stent from the arteries of young children, particularly in the case of distal occlusion as in one of our two cases, to us, carries a higher risk of hemorrhagic complication compared to direct aspiration. Distal anchoring by the stentriever in the occluded artery increases the risk of straightening the parent vessel and causing traction on perforators with risk of subsequent SAH. In direct aspiration, as there is no distal anchoring, the risk should be lower.

The positive outcomes observed in our patients, including successful recanalization and absence of complications, support the feasibility of MT in infants and align with findings from prior studies. This indicates that pediatric patients might benefit from MT [3, 4, 5, 11], provided the operating neurointerventionalist has experience with pediatric cases. The technical challenges of performing MT in infants, such as navigating the small and fragile vessels, were successfully managed in our cases using thromboaspiration for clot removal. We endeavored an extremely cautious treatment, as demonstrated by the length of intervention (occlusion to recanalization time was 116 and 41 min, respectively). Our team is involved and well experienced in neuro‐endovascular therapy of children, especially neonates. Based on our experience, we're all very much in favor of thrombectomy in children, whatever their age, as this disease is potentially devastating at any age. But with the higher risk of ischemic‐hemorrhagic complications in this population, an inexperienced team might not achieve the same results, especially for children under 1 year. In the case of an inexperienced team, we can't make any suggestions, and such intervention should be indicated with great caution, after an accurate assessment of its own skills. Optimization of medical management should be considered as the first line of treatment. As in all cases of acute stroke, at least antiplatelet therapy with aspirin 3–5 mg/kg should be considered, if available. IV or IA thrombolysis or anticoagulation should be considered on a case‐by‐case basis. Our cases differ significantly from previous reports due to the unusual circumstance of thrombosis occurring during chemotherapy administration. In both cases, the etiology was clearly identified (thromboembolic complication), which might not be the case in a pediatric stroke. Physicians should be aware that in acute pediatric stroke, the etiology must first be identified to decide how to proceed with thrombectomy. Indeed, vessel arteriopathy (focal dissection, focal post‐infectious arteriopathy) is very frequent, affecting about one‐third of children diagnosed with arterial ischemic stroke [12]. Often, these arteriopathies do not involve a true clot inside the lumen, which decreases the chance of recanalizing the vessel and increases the risk of vessel damage during the procedure [13].

Of course, we cannot draw conclusions from this very small case series, and our findings emphasize the need for more data in young children. The current lack of comprehensive data and therefore guidelines highlights the critical need for further research and collaboration in this field.

## Author Contributions

**Erensu Mengüşoğlu:** conceptualization, formal analysis, investigation, writing – original draft, writing – review and editing. **Francesco Puccinelli:** investigation, writing – original draft, writing – review and editing. **Steven D. Hajdu:** investigation, writing – original draft, writing – review and editing. **Bruno Bartolini:** investigation, writing – original draft, writing – review and editing. **Guillaume Saliou:** conceptualization, formal analysis, investigation, supervision, writing – original draft, writing – review and editing.

## Consent

Written informed consent was obtained from the legal guardians of both patients for the procedure and the publication of this case report.

## Conflicts of Interest

The authors declare no conflicts of interest.

## Data Availability

The data that support the findings of this study are available from the corresponding author upon reasonable request.
